# Using the Living Lab Methodology to Build Inclusive Communities: A Participatory Action Research

**DOI:** 10.1002/jcop.70054

**Published:** 2025-11-04

**Authors:** Fortuna Procentese, Flora Gatti, Francesca Margherita De Falco, Yuri Autorino, Biagio Marano

**Affiliations:** ^1^ Dipartimento di Studi Umanistici Università degli Studi di Napoli Federico II Naples Italy; ^2^ Dipartimento Chirurgico, Medico, Odontoiatrico e di Scienze Morfologiche con Interesse Trapiantologico, Oncologico e di Medicina Rigenerativa Università degli Studi di Modena e Reggio Emilia Modena Italy

**Keywords:** living Lab, local community, participatory action research, responsible togetherness, social inclusion, social innovation

## Abstract

Living Labs (LLs) have spread as a methodology for the development of innovative perspectives. It has mainly been used in the Information and Communication Technologies (ICTs) field, but less in the social sciences. The present study deepens the use of this methodology in the psychosocial field within the EU‐project YouCount, with the aim of developing innovative processes enhancing youth social inclusion. Participants of the LL were 30 Italian citizens aged between 22 and 73 (*M* = 31.63; *SD* = 11.67). 14 were referents of local associations and social groups, representing seven different realities dealing with migratory processes and social inclusion issues; the remaining participants were young local and migrant youths civically and socially active. During the LL, several participatory methodologies (e.g., World Café, Co‐design sessions, video‐interviews, video‐making) were used to facilitate the unfolding of social innovation and collaborative processes among participants. Through this path, new ways of living together in the community emerged, based on the reciprocal acknowledgment and collaboration among participants for the bottom‐up development of common planning aimed at supporting local services.

## 1 Introduction

The European Network of Living Lab (ENoLL) defines Living Labs (LLs) as “open, user‐centered innovation ecosystems, based on a systematic co‐creation approach, that integrate research and innovation processes in communities and real‐life contexts” Piotrmill 2023); based on this, LLs are recognized as processes able to develop social innovations by the European Commission (2006). These definitions emphasize LLs as physical and relational spaces in which confrontation is built in a different and innovative perspective, and as open innovation environments where the co‐creation process developed by participants produces new services and social infrastructures and represents one of the main results itself (Dekker et al. 2020).

In the field of social innovation, LLs represent a methodology allowing to explore several categories of tangible and intangible needs emerging from the participants, based on the acknowledgment that the latter are privileged witnesses of the challenges they experience every day and the LLs deal with (Viswanathan et al. 2004); the overall aim is to modify the daily interaction between users and their living contexts (Dell'Era and Landoni 2014). Within LL paths, the shared responsibilities for the identification of different solutions translate into the co‐creative process; even though professionals facilitate the interactions during the meeting, participants are free to interact and co‐create in full autonomy (Dell'Era and Landoni 2014). This way of co‐construction—which leads to possible solutions to a problem by adopting an abductive logic (Kolko 2011)—is also referred to as user‐driven innovation (De Moor et al. 2010). Based on this, LLs fit within Participatory Action Research (PAR) approach, sharing its basic premises—that is, the focus on citizens' active involvement in the promoted processes, aimed at addressing and solving social problems affecting their lives to improve individual and collective daily life conditions (Cammarota and Fine 2008).

Therefore, although LLs originated in the field of Information and Communication Technologies (ICTs) innovation, in recent times they have been increasingly used in the field of social sciences and humanities, for the promotion of public and social innovation too (Dekker et al. 2020). In this field, LLs have been implemented as research and development processes of innovation unfolding in real‐life settings and foreseeing the collaboration among several different stakeholders as co‐creators of such processes (Dekker et al. 2020). For example, some studies have addressed urban and rural LLs, which were implemented by local governments and involved citizens in the development of collaborative answers to local problems related to participative governance arrangements (e.g., Bifulco et al. 2017), sustainability (e.g., Voytenko et al. 2016), urban planning (e.g., Lehmann et al. 2015). In other experiences, the use of LLs was aimed at the promotion of technologies use in public administration within processes such as e‐government and e‐participation (e.g., Kareborn and Stahlbrost 2009; Cleland et al. 2012; Edwards‐Schachter et al. 2012; Galiano et al. 2014). In the same vein, LLs were implemented for the promotion of social inclusion processes across Europe within the EU‐project YouCount (Pataki et al. 2023a)—which is the focus of the present paper.

Therefore, LLs can be used to encourage interaction and confrontation between stakeholders and citizens who have common goals but different cultures, representations, and experiences (Pataki et al. 2023a). They hold potential to enhance local communities' capacity to address and solve challenges internally (Hooli et al. 2016)—that is, they can represent not only socio‐technical innovations hubs but also catalysts for building community problem‐solving capabilities. In this vein, active participation in LLs can be a vehicle for consolidating responsibility‐taking and ‐sharing processes in the community, as the latter organizes and takes action to address local issues, promoting more responsible styles of togetherness (Procentese et al. 2011; Procentese and Gatti 2019b).

Thus, LLs represent physical but also relational spaces (Butkevičienė et al. 2021) in which collaborative processes can unfold by involving both professional researchers and end‐users—in the field of humanities and social sciences, the latter are stakeholders and citizens—in continuous discussion sessions which can be shaped and led relying on specific methodologies; the aim is to meet and solve shared needs, create opportunities, empower participants so that their transformative attitudes and actions can modify the current social dynamics and structures (Pataki et al. 2023a).

Based on this, the present work aims at deepening the unfolding of collaborative processes through LL meetings to foster responsibility‐taking and ‐sharing processes in the community, up to co‐creating new strategies to manage local issues along with participants. Specifically, it addresses the LL path developed by the Italian research team within the project “YouCount ‐ Empowering Youth and Cocreating Social Innovations and Policymaking through Youth‐Focused Citizen Social Science” (from now on, “YouCount”), a Citizen Social Science (CSS) project funded by the European Union under the Horizon 2020 program, as a case study. Attention will be paid to the innovative processes and social dynamics unfolding during such LL, as well as to the methodologies used and to the materials produced. The main aim of the described LL was to foster the social network between different stakeholders—such as associations, cooperatives, social enterprises, and young citizens—who were all interested in enhancing social inclusion processes for youths with migratory background living in the second and third municipalities of Naples (Italy). Through the LL, an extended dialogue, the sharing of perspectives, and confrontation processes between the participants were promoted, resulting in shared and feasible paths aimed at fostering social inclusion processes in the targeted neighborhoods. Indeed, empowering the social groups that are usually excluded from decision‐making processes and promoting intercultural dialogue between groups of different nationalities or with different social and professional skills are fundamental objectives for institutions, local associations, and social services promoting more inclusive ways of living together (Cornwall 2008; Sampedro and Camarero 2018).

## 2 Local Active Participation Fostering Responsible Styles of Togetherness

Researchers, policymakers, and practitioners endeavor to understand how people from different cultural backgrounds and social groups can—or should—live together in the same social context (Sam et al. 2013) due to the complexities characterizing modern local communities (e.g., Gatti et al. 2023; Procentese and Gatti 2022). Indeed, the spread of values focused on reciprocal distrust and defense rather than reciprocal knowledge and sharing is producing increasingly complex forms of local social togetherness (Natale et al. 2016; Procentese and Gatti 2019a; Procentese et al. 2011; Tonkiss 2003).

In this context, the promotion of community participation is crucial in addressing social challenges, increasing immigration, recent economic crises, and social and political stratifications—which all represent issues have the potential to create tensions among different ethnic, social, and cultural groups (Landis and Albert 2012). Participation in the local community of belonging encompasses all the shared activities individuals engage in to improve their living conditions (Adler and Goggin 2005), such as volunteering and activism, but also civic, social, and politic involvement. Citizens' engagement and participation bring benefits to both individuals and institutions (Montero 2004), fostering social cohesion and well‐being at both individual and collective levels (Cantor and Sanderson 1999; Chan et al. 2006; Clary and Snyder 2002; Delhey and Dragolov 2016; Hyman 2002). They can be particularly positive for youths and individuals not being well integrated into their social context (Lawton et al. 2021; Piliavin and Siegl 2007). Indeed, according to the theory of collective action and social change (van Zomeren et al. 2008), involving members of disadvantaged groups in collective actions aimed at reducing inequalities can enhance their group effectiveness (Dixon et al. 2017).

The promotion of local generative and co‐creative meetings represents a possible answer to the need for interventions aimed at fostering new ways of living together within local communities and rebuilding individuals' sense of responsibility toward them (Arcidiacono et al. 2007; Procentese et al. 2011). Indeed, creating spaces and opportunities to foster dialogue among different cultures and social groups, open communication, and constructive engagement is essential for promoting mutual understanding, interculturality, and social inclusion (Juvonen et al. 2019; Pienimäki 2021; Sampedro and Camarero 2018). These efforts build upon citizens' representations about how to live together in the community and work together to improve it through responsibility‐taking processes, fostering citizens' tie to their community and engagement for it at the same time, defining an active and engaged citizenship (Procentese and Gatti 2022; Zaff et al. 2010).

In this vein, activating participatory paths that actively involve and engage citizens with different cultural backgrounds and various territorial actors in the community represents a strategy to promote more responsible styles of togetherness. This is rooted in recognizing the pivotal role of participation in enhancing the sense of responsible togetherness (SoRT) for one's community (Procentese and Gatti 2019b), emphasizing the quality of the ties towards and within the community as elements through which communities mobilize for social change (Procentese and Gatti 2019b). Therefore, these interventions can focus on creating synergies among local actors—that is, associations, institutions, citizens—and fostering collaboration between associations and institutions, as it was the case for the Italian LL experience within the YouCount project.

## 3 Theoretical and Methodological Aspects of Living Labs

The LL is a methodology centered on participants and real‐life contexts (Kareborn and Stahlbrost 2009; Følstad 2008), in which the former co‐create the concepts to be addressed during the LL based on their own experiences and evaluations (Bekker and Long 2000). Two aspects specifically make LLs differ from other innovative methods, that is, their high realism and the degree of involvement: indeed, users became part of the innovation themselves (Schuurman and De Marez 2012). Such involvement occurs using physical artefacts that work as thinking tools aimed at improving practices in constantly changing real‐world settings (Dell'Era and Landoni 2014; Eriksson et al. 2005; Romero 2017; Schuurman et al. 2012).

According to Westerlund and Leminen (2011), LLs are composed of four key actors: enablers, providers, users, and utilizers: “enablers refer to the organizations that make it all possible, those that enable the activities of living labs and support them by promoting them or allocating financial backing or space for living labs. Enablers could be public actors, financiers, or nongovernmental organizations (such as towns), municipalities, and regional development organizations. Providers, meanwhile, are development organizations such as educational institutes, universities, or consultants that bring knowledge and expertise, as well as innovation support activities. Users represent the citizens or end customers, and they are active or passive actors that participate in living labs in various roles. Finally, utilizers are the public or private organizations that will benefit from the results of innovation activities in many ways” (Hossain et al. 2019, 981).

In the field of humanities and social sciences, LLs can also be defined as open community spaces where different local actors collaboratively interact, transforming challenges within the communities into new opportunities for innovation (Hooli et al. 2016). They fit in the tradition of PAR, as they aim to produce scientifically and socially sound knowledge and transformative actions (Bartels and Wittmayer 2014; Huxham 2003; Orefice 2006) through involving relevant stakeholders. Indeed, within this perspective, individuals and communities being affected by a given social issue are reckoned as the privileged witnesses to be involved in the planning and implementation of actions aimed at solving them (Viswanathan et al. 2004). In the same vein, the setting of implementation is reckoned in real‐life settings wherein the product to be co‐created is needed and will be implemented—that is, they are context‐related processes (Dekker et al. 2020; Viswanathan et al. 2004).

Therefore, LLs are based on a participatory, democratic approach valuing citizens' direct participation as able to generate more effective and sustainable results, emphasizing the importance of listening to a plurality of perspectives, knowledges, and needs to properly address territorial challenges (Elliott 1991; Francescato et al. 2002) and highlighting the importance of constant reflexivity as circumstances unfold (Kareborn and Stahlbrost 2009). Indeed, a prerequisite for positive social impact is the adoption of a model based on dialogic rather than linear schemes (Karrsen & Larrea 2016). In this vein, LLs require “an iterative process of gradually improving and refining a product in successive stages of research and design” (1210) and the final result of such process is not set from the beginning (Dekker et al. 2020).

As LLs are aimed at transforming communication models and empowering communities to successfully address specific territorial challenges and promote sustainable and inclusive development (Karrsen & Larrea 2016), five key principles represent the basis of this methodology (CoreLabs 2007) making it relevant in the field of social sciences and humanities too: (a) continuity, (b) openness, (c) realism, (d), empowerment of the users, and (e) spontaneity. Indeed, innovation processes should be as open as possible to gather many different perspectives; however, good cross‐border collaboration—which strengthens creativity and innovation—builds upon trust, which takes time to develop (Kareborn and Stahlbrost 2009; Chesbrough 2003; Chesbrough and Appleyard 2007). Furthermore, an open process allows to support the co‐creation of user‐driven innovations, including all the interested users wherever and whoever they are. Indeed, another key issue refers to bringing enough power to the participants (Kareborn and Stahlbrost 2009) and to the community as a whole (Hooli et al. 2016) to achieve rapid progress. Focusing on real users and facilitating as realistic as possible situations and behaviors allows to achieve results that could be more valid and expendable in real‐life contexts and experiences—which is what distinguishes LLs from other kinds of open co‐creation environments. Last, to succeed with new innovations, it is important to inspire usage, meet personal desires, and fit and contribute to societal and social needs. Here, the ability to detect, aggregate, and analyze spontaneous users' reactions and ideas over time plays a key role (Kareborn and Stahlbrost 2009).

Therefore, the main role of LLs is “to engage and empower users to participate in the generation of valuable and sustainable assets toward objectives set up by its partners and customers” (CoreLabs 2007, 9). Based on this, LLs can represent a *territorial agora* (Karlsen and Larrea 2014) where different territorial actors meet, scientific and social problems are defined, and potential solutions are negotiated (Nowotny et al. 2001; Schwandt et al. 2007); it represents an interconnected reality, shaped by the interactions among the involved actors through continuous dialogue (Karlsen and Larrea 2014). These processes foster the coproduction of concrete solutions to territorial challenges by combining academic and experiential knowledge through a wide pool of methodologies and tools (Dekker et al. 2020).

Overall, professional researchers play the role of agents of change, collaborating with the territorial actors involved in the unfolding processes (Karlsen and Larrea 2014). Indeed, another characteristic of LLs which becomes particularly relevant in the field of humanities and social sciences is the involvement of stakeholders during the process, and the partnership between private, public, and people (Veeckman et al. 2013).

## 4 LL Use in the Italian Experience Within YouCount

### 4.1 The Context: The YouCount Project

The YouCount project (February 2021–January 2024; https://cordis.europa.eu/project/id/101005931) aimed at co‐creating new knowledge and social innovations to increase the social inclusion of different groups of marginalized youths (aged between 16 and 30) across Europe. Youth social inclusion was meant as a multidimensional process referring to the opportunities and resources favoring participatory processes, but also the relational, economic, and political characteristics of a specific social context (Procentese and Gatti 2024). To work towards project aims, 10 local cases were implemented in 9 European countries by developing two parallel paths each (Pataki et al. 2023b; Ridley et al. 2022): (a) working with citizens to foster different and more inclusive ways of living their communities of belonging; (b) setting up local LLs involving stakeholders, young citizens, and institutional referents to identify together different paths towards more inclusive communities. Collaboration and co‐creation of knowledge and social processes aimed at promoting social inclusion were key elements in project unfolding (Procentese et al. 2024; Ridley et al. 2023).

Within this overall framework, LLs were specifically implemented as experiences aimed at promoting processes enhancing local participation, individual and shared responsibilities (Frascaroli et al. 2016; Natale et al. 2016), sense of community (McMillan and Chavis 1986; Procentese et al. 2019), and sense of responsible togetherness (Procentese and Gatti 2019b).

### 4.2 Outline and Aims of the LL

The Italian LL experience took place in Naples between April and September 2022— consistently with LLs having a medium‐ to long‐term time span (Dekker et al. 2020; Følstad 2008).

It shaped as a series of eight meetings (lasting 2 h each) aimed at fostering dialogue between local stakeholders and citizens representing different professional and cultural visions in local communities hosting young people with a migratory background; in this vein, it was an *incubator of inclusive ideas* aimed at promoting different ways of living together and experiencing local social relationships. The shared view leading the development of future joint projects during the LL built upon the need to develop empowering processes and detect the opportunities for community members to meet and match about the topics and issues related to social inclusion issues and how to deal with them like a *territorial agora* (Karlsen and Larrea 2014).

Proposals for concrete actions to be jointly carried out were shared and discussed from a bottom‐up perspective, taking into account citizens' and stakeholders' real needs. Beyond the specific ideas developed, the opportunity to meet and match allowed participants to feel part of a group planning collective and shared actions, which is an experience fostering social inclusion processes. Consistently, side aims were the promotion of a stronger social network among local associations and stakeholders, as well as the involvement of citizens and stakeholders in the joint planning of activities aimed at promoting different ways of living together in local communities and sharing the local social and physical context.

The first to the fourth LL meetings consisted in three phases: the first and the last one unfolded in a unique group—that is, they were plenary sessions—while the second one required participants to work in small groups. During each of these meetings, participants were first introduced to the discussion by listening to a short summary of the main ideas and suggestions emerged during the previous one; then, the stimulus questions were described by professional researchers, and participants were asked to work in small groups around them according to the methods described. Dividing participants into small groups allowed richer and more participated discussions, as each participant had the chance to express their viewpoints and feel heard by others. The small groups did not have a fixed composition—that is, the participants in each small group were grouped differently each time; professional researchers paid attention to balancing the small groups with reference to participants' gender, role (i.e., stakeholders/referents of associations vs. youths), and ethnic background (i.e., Italian or migrants).

Then, the fifth meeting was aimed at participants and members of the research team co‐creating a short video aimed at presenting the LL experience, what it meant to its participants, and the main benefits they got from it. The sixth meeting was held at the Trianon Viviani Theater (in Forcella, a neighborhood in Naples) and represented an opportunity to engage with more interested citizens and to share the LL experience from participants' perspective by showing the video they co‐produced. The last two meetings were aimed at systemizing the ideas and plans stemming from the LL experience and from the meeting at the theater to identify the contribution each one was able to provide at that stage along with the stakeholders and the youths.

All meetings but the sixth were held in the same venue, a room in a venue of the University of Naples Federico II, located in the city center in Naples. The room was chosen based on it having movable chairs that would allow for frontal or small group work settings as needed.

Constant meetings involving researchers and co‐researchers were held between one LL meeting and the next, to reflect together and discuss the progress of the process and the content emerging from time to time (Kareborn and Stahlbrost 2009), so as to better calibrate the activities and stimuli to be proposed during the next meeting, in line with PAR main principles (Bartels and Wittmayer 2014; Cammarota and Fine 2008; Huxham 2003; Orefice 2006). The choice of PAR and a collaborative approach is consistent with the Community Psychology approach—that lead the choices made—and with its main pillar of making people aware, capable of handling tools, aware of their conditions, needs, potential, resources, limits, values, and desires as protagonists of the local community, and able to read the processes while they happen—that is, of enabling them to be an active part of the unfolding process of change (Arcidiacono and Marta 2008).

Consistently with the aims of the LL, the methodologies and tools used during the meetings were participatory ones: (a) Word Café (first meeting), and brainstorming and group discussion (second and seventh meetings) sessions aimed at understanding participants' viewpoints and promoting reflections on the factors that positively and negatively impact social inclusion, as well as on the collective actions that can foster it; (b) several, subsequent Codesign sessions (third and fourth meetings) aimed at identifying and planning innovative paths to promote local social inclusion, together with the citizens and starting from their experiences and perspectives (c) video‐interviews and videomaking (fifth meeting), aimed at sharing the LL experience with the broader community moving from participants' viewpoints. The process was meant as iterative, with participants continuously working on their ideas and perspectives up to defining the final product (Dekker et al. 2020). The goal was to put participants in the position to plan together activities and processes favoring opportunities for local and migrant citizens to meet and get to know each other better. Indeed, to achieve a different and more responsible way of living together within local communities, citizens need moments to share their viewpoints and understand others', respecting individual differences rather than denying them (Procentese et al. 2011). A summary of the LL process (aims, methodologies, and main results for each meeting) is in Table 1.

**Table 1:** Summary of the LL process: aims, methodologies, and main results.

Meeting (Duration)	Aims	Methodology	Main results
1 (2 h)	Deepening (a) views and experiences about youth social inclusion and (b) suggestions to enhance it	Word café	Identification of participants' perspectives and experiences about youths' social inclusion experiences, migratory processes, and local social networks.
2 (2 h)	Gathering perspectives, ideas, and potential initiatives aimed at promoting the shift from the actual, welfarist vision to a reciprocity‐based one	Brainstorming and group discussion	Identification of participants' ideas and suggestions about how to foster this cultural shift through social opportunities, gatherings, and events.
3 (2 h)	Co‐designing a proposal of activities (aims, participants, actions and activities, phases and timing, sustainability) to be implemented to foster a more inclusive style of togetherness	Codesign session	A co‐created proposal for joint activities aimed at allowing local and migrant youths to better know each other by taking advantage of several expressive tools (e.g., language, colors, gestures).
4 (2 h)	Addressing the feasibility of the chosen proposal	Codesign session	Editing a short video about LL aims, experience, and learnings from participants' viewpoint was selected as a strategy to advertise the proposal to be developed and enhance its feasibility.
5 (2 h)	Producing the materials and editing the video	Video‐interviews and videomaking	Materials (video‐interviews, introduction, symbolic scene, “Vocabulary of Inclusion”) for the video were produced; the video was edited.
6 (2 h)	Sharing the LL experience with the broader community	Public meeting and exhibition	The video was shared with the broader community, along with other materials produced during the project unfolding; LL members and citizens from the broader community shared their experiences and perspectives about social inclusion, intercultural processes, and LL experience – if involved.
7 (2 h)	Systemizing the ideas and future plans from LL experience and the meeting with the broader community	Brainstorming and group discussion	Identification of future plans and projects; definition of the contribution each one was able to provide at that stage for their development and actual implementation.
8 (2 h)	Wrapping up and planning future steps	Group discussion	Definition of the contribution each one was able to provide at that stage for the development and actual implementation of future joint plans and projects.

Abbreviation: LL, Living Lab.

#### 4.2.1 World Café

The Word Café is a participatory research method “aimed at producing authentic and collaborative conversations about real‐life topics that matter to participants (Brown 2002; Brown and Isaacs 2005; Schieffer et al. 2004)” (Gatti and Procentese 2021, 3). Participants bring different ideas and viewpoints, which are made explicit and shared with the group during the meeting. Overall, “it allows building knowledge by relying on the active involvement of the participants in a process of social interaction and co‐construction of awareness that is aimed at producing a change” (Gatti and Procentese 2021, 4). Through this, the Word Café methodology allows to detect feasible paths aimed at improving the current circumstances with reference to a topic building on open dialogue, confrontation, and exchanges among participants.

Usually, each World Café session comprises several rounds led by different questions referring to the same overall topic, since this is considered a useful way to address more inter‐related topics while avoiding participants to get tired (Weitzenegger 2010); these questions allow participants to explore new ideas about the main topic and to progress towards always more concrete issues about it (Brown and Isaacs 2005).

The participants are divided into small sub‐groups by the leaders of the activity to make them discuss the various questions; all the sub‐groups discuss the same question at the same time. During the first round, a participant for each table is democratically chosen by the others in each sub‐group to become the *host* and remains at the table, while the others are asked to change table at the end of each round to *cross‐pollinate* ideas and produce new connections across the sub‐groups (Gatti and Procentese 2021). At the start of each new round, the hosts share with the newcomers to their table what was discussed by the previous participants, so that the conversation can continue from where it stopped, advancing toward always deeper exploration of the topics; this is repeated for each round. This allows participants to feel that their ideas are part of a bigger, common, process (Brown and Isaacs 2005). At the end of the last round, each group is asked to identify five keywords, summing up the whole discussion held through the different rounds at that table. Then, the keywords are shared in the wider group using them as dominos. This provides further opportunities to debate, produces shared knowledge, and sets common goals among all participants (Brown and Isaacs 2005).

Within the described LL, Word Cafés were used to foster the exchange of viewpoints and the emergence of shared paths for the enhancement of social inclusion processes through the increase of socialization spaces and opportunities for local and migrant youths. The Word Café format made it possible to bring out individual attitudes, motivations, and beliefs about social inclusion: referents of associations and stakeholders shared their past and current activities and plans for the promotion of social inclusion, while local and migrant citizens shared their experiences from their viewpoints.

#### 4.2.2 Brainstorming and Group Discussions

The brainstorming (Martini and Sequi 1988) allowed to prompt a deep and co‐created group discussion, with participants engaging in reflections and dialogues about social inclusion and migratory experiences. During each brainstorming and group discussion session, post‐it and pieces of paper were used to take note of the ideas stemming; these materials were used as support when sharing the contents emerged in the plenary sessions.

Overall, having brainstorming and group discussion sessions involving local citizens, citizens with a migratory background, and stakeholders dealing with this topic represented a first opportunity for different cultures to meet and match, to exchange viewpoints, and to share experiences and perspectives about their social inclusion, consistently with the aims of the LL.

#### 4.2.3 Codesign Sessions

Codesign sessions were held as a means to make participants think about social inclusion processes and how to promote them with specific reference to native and migrant youths. These sessions represented opportunities for deep collaboration among participants, who were asked to work together to identify and define innovative paths aimed at promoting youth social inclusion locally based on their own viewpoints, experiences, and feelings.

Each codesign session was introduced by a brief summary of the results from the previous meeting; then, participants were divided into small sub‐groups by the professional researchers. All the sub‐groups moved from the same stimuli to proceed towards always more concrete ideas, activities, and plans to be jointly implemented. At the end of each session, some referents of each sub‐group shared the main topics and ideas produced by the group in a plenary session, and all the participants discussed the shared contents up to reaching an agreement on a unique set of ideas. Such set of ideas would have represented the starting point for the following codesign session.

#### 4.2.4 Video‐Interviews and Videomaking

As an attempt to spread the positive experience of the LL and the contents emerged during it, participants worked together to the production of the materials and scenes to be used to build a video about their LL experience and their shared viewpoints about social inclusion processes in Naples. The script, contents, recordings to be used, as well as the aspects that should have emerged from the video, were selected by the participants along with the research team.

Short video‐interviews—based on one or two questions—were administered to LL participants by the research team as a shared endeavor to gather visual witnesses of the implemented path moving from words and experiences of those who were part of it. Takes by these videos and photos of the materials produced during the meetings (post‐it notes and proposals for future actions and paths to be co‐developed) and of the meetings themselves were used for producing a short video summarizing the LL experience (Arcidiacono et al. 2016). This allowed to share the products and processes characterizing the LL experience with a broader audience, intending to catch the interest and involve more citizens and stakeholders dealing with social inclusion and local togetherness issues in the targeted area.

#### 4.2.5 Public Meeting and Exhibition

A public meeting was held in the form of an exhibition as an endeavor to engage with further interested citizens from the target community (Arcidiacono et al. 2016). The meeting was aimed at sharing the LL experience building on participants' perspective: materials (photos, texts, the video) from the previous meetings were gathered by the participants along with the researchers, and a lineup for the whole meeting was agreed upon. The rationale for the lineup was to allow participants to share the processes they engaged with, and the outcomes achieved as the whole process unfolded and became more complex. Therefore, participants were engaged at the forefront of selection of the contents to be used, based on the idea that their choices would have been *performative* in nature (Holm 2008), in that they represent what they want to show with respect to a given theme or process, that is, what they consider relevant to that theme.

Indeed, pictures and videos can increase awareness among citizens by creating opportunities to share contents and goals within groups (both the one creating them and the one taking part in the exhibition), through their production and discussions of the meanings they evoke. By doing so, they make it possible for the community to assess available resources and possible obstacles with reference to an issue, activating processes serving individual and community changes based on these reflections (Wang 2006). Additionally, such processes bring out new knowledge with respect to the topic and how it can be intervened upon from the experience of the participants themselves, through the meanings they evoke (Arcidiacono et al. 2016).

### 4.3 Methods

#### 4.3.1 Participants

The Italian LL involved 30 participants aged between 22 and 73 (*M* = 31.63; *SD* = 11.67); most of them were female (*n* = 20). Based on the ecological nature (Bronfenbrenner 1979; Lewin 1951) of the project—as well as of social inclusion processes at large—participants included both referents of local associations and youths from the target local community. Specifically, 14 participants were referents of local associations and social groups, representing seven different realities dealing with migratory processes and social inclusion issues in the targeted local area; the remaining participants were young local and migrant youths civically and socially active around these topics or interested in becoming so. However, it is to mention that several referents of local associations and social groups were youths too—as proven by the average age—and had migratory background in some cases. Participants having migratory background were French, Tunisian, or from other African countries—they chose not to specify which ones.

Participants were invited to take part in the LL meetings by sending them a flyer including the title, the dates, and the venues for the meetings as a form of invitation. Indeed, in a preliminary phase, the local research team had mapped the targeted area of the second and third municipalities of Naples with the aim of identifying the associations and social groups being locally active and dealing with migratory processes and social inclusion. Attending all meetings was highly recommended but it was not compulsory to take part in the LL process; therefore, some participants joined after one or two meetings from the beginning of the LL and others missed a few meetings during its course; on average, between 15 and 20 participants attended each meeting. When attending their first meeting, participants were asked to express their written informed consent to take part in the LL process and allow the research team to anonymously use the materials that would have been produced within it by filling in and signing a form.

#### 4.3.2 Data Collection and Analysis

The data gathered within the LL experience were: (a) the materials produced during the meetings (memos, post‐it, notes, billboards, keywords, videos); (b) the contents emerging during the meetings; (c) researchers' and coresearchers' logbooks related to the meetings—in terms of both contents and unfolding processes.

Overall, these data were analyzed and deepened with the aim to untangle: (a) participants' perspectives about social inclusion processes, experiences, drivers, and obstacles; (b) potential paths toward more inclusive communities according to them; (c) whether—and how—LL experience could serve as a catalyst for more cohesive and inclusive communities itself due to its intrinsic participatory and collaborative nature. The analysis was carried out through constant confrontation and discussion among researchers and co‐researchers within the meetings (Kareborn and Stahlbrost 2009) happening between a LL meeting and the following one; during these internal meetings, reports were produced to keep track of the contents, decisions, and processes. Such reports were included in the analysis themselves, as they could provide further data with reference to the unfolding processes and dynamics. Therefore, triangulation of investigators carrying out the analysis and data to be analyzed were applied (Lincoln et al. 1985; Schwandt et al. 2007); furthermore, the analysis represented a process unfolding throughout the whole LL experience, so that member‐check strategies could be used to further verify the meanings and links attributed to the emerging concepts and processes (Lincoln et al. 1985; Schwandt et al. 2007).

Overall, the intertwining of the materials produced, the contents emerged, and researchers' and co‐researchers' content‐ and process‐related considerations allowed to address the complexities underlying these topics, while taking also into account the participatory and collaborative nature of LL experience (Hooli et al. 2016) and the role of researchers as agents of change along with LL participants (Karslen & Larrea, 2014).

### 4.4 Results

Overall, the main results stemming from the described LL experience (see Table 1 for a summary of the main aims, methodologies, and results from the LL meetings) refer to (a) the views and knowledge that were shared within the group about social inclusion processes, (b) the jointly planned paths towards more inclusive communities, and (c) the process unfolding across the LL meetings, which allowed to strengthen the local social network involving citizens and stakeholders dealing with the topic.

Indeed, at the very start of the LL, after participants introducing themselves to others to promote the creation of a glued group, a World Cafè session was used to deepen (a) their views and experiences about youth social inclusion and (b) their suggestions to enhance it. From this meeting, two main learnings stemmed, representing the basis for future ones. During this meeting, a potential obstacle was related to some participants speaking different languages due to their different backgrounds; based on this, researchers paid attention to how to facilitate communication and exchanges within the group also by overcoming language barriers if needed: translating in English or asking other participants for help in translating when a different language was needed—so that no one was left out from the unfolding processes—were chosen as the best solutions.

As to the relationships among participants, a clear lack of reciprocal knowledge and acknowledgment emerged despite them including stakeholders working on the same issues and in the same area; local relationships among stakeholders were described as scarce and not glued, so that they were physically close to each other due to them working in the same local area yet not collaborating towards shared aims despite this. This significantly reduced their capacity for advocacy—that is, to influence public policies and resource allocation within political, economic, and social systems and their institutions. One of the participants (a referent of a local association dealing with social inclusion issues) described the local social network as an archipelago that has yet to compact: “*the archipelago for me is a split but at the same time united element, it is a whole made up of many different small components represented by the many islands, different in customs, organization, and perhaps economic and political structure. So, we are like islands to be compacted, but each remaining in our uniqueness: the islands are fixed and immobile, we cannot move them or change their geographical appearance, rather we can visit them, we can join the customs of their inhabitants, we can experience them in the diversity that distinguishes them from our native culture, we can accept them*”. This could represent an additional obstacle to making participants work together in a collaborative and participatory way during LL unfolding, yet it did not in the end. Indeed, the metaphor of the archipelago recalled the idea of stakeholders being close yet different and not connected to each other despite the common aims, but also implied the opportunities brought about by further processes of reciprocal knowledge and acknowledgment, which could allow to experience and accept diversity as a resource.

In addition, as to the views about social inclusion and migratory processes, a spread welfarist perception of migrants as “*needing help*” emerged as characterizing the broader community, standing against the need to understand that migrants are people, have power, and have their own experiences, history, and resources—as acknowledged by all the participants, be them stakeholders or young citizens. As underlined by the participants, a different and more balanced vision should rather reckon everyone′s resources, power, and experiences as an added value to be included in building a new, more equal daily life. In this vein, a bidirectional perspective about the relationship between locals and migrants (i.e., considering social inclusion as a two‐way process; Pienimäki 2021; Sampedro and Camarero 2018) stems as something to be implemented, against the actual, unidirectional one.

Based on this, since the very first meeting—and throughout all the following ones—the main focus spontaneously revolved around the need to promote the acknowledgment of migrants not only as beneficiaries who need the help and support of local services, but also as people who have characteristics and a potential to unfold, and who can equally contribute to the well‐being of the community. Therefore, the following main needs emerged: (a) to rethink and rebuild a language so that it could be more inclusive (e.g., according to participants, the word “migrant” is to be favored over “foreigner”); (b) to give centrality to migrants and to the narration of their experiences, to valorize them; (c) to spread curiosity towards and listening to extraneousness and differences; (d) to deconstruct of implicit and explicit prejudices, to reduce discrimination; (e) to acknowledge everyone′s needs and the possibility of mobilizing personal and collective resources to satisfy them.

Moving from this, from the second meeting on participants worked on putting together perspectives, ideas, and potential initiatives aimed at promoting the shift from the actual vision to the bidirectional one. The emerging ideas revolved around some main themes across which the potential of local social gatherings for social inclusion processes clearly stems: (a) promoting social opportunities in which citizens from different ethnic backgrounds can get to know each other, countering the already existing prejudices and (self‐)ghettoization processes; (b) viewing social gatherings as further opportunities for migrants to better learn Italian in an informal social context; (c) promoting initiatives and events aimed at migrants introducing their cultures, traditions, and habits to Italian youths, so that the latter can experience and better understand them.

Based on the ideas emerged, participants were asked to jointly co‐design a feasible proposal of activities to be implemented towards these goals by outlining the aims of their proposal, the participants to be involved, the actions and activities to be concretely implemented, the needed phases and timing, and the sustainability of the project. Overall, two main ideas about how to foster a different and more inclusive way of living together within local communities emerged, and participants were asked to discuss them to reach an agreement about one shared proposal. In the end, according to the dialogic and democratic process of the LL (Elliott 1991; Francescato et al 2002; Karrsen & Larrea 2016), participants spontaneously merged the two ideas into a unique, more complex one, which incorporated the main points of both in a path of activities and initiatives aimed at allowing local and migrant youths to better know each other taking advantage of several expressive tools (e.g., language, colors, gestures).

Then, the feasibility of the emerging proposal was specifically addressed. During such discussion, one of the stakeholders suggested co‐producing a short video narrating the LL aims, experience, and learnings from participants' viewpoint as a starting point for catching the interest of more young citizens and advertising the proposal to be developed. Since the whole group—that is, researchers and participants—agreed about this being a good idea, participants were asked to think about how to develop the video, what to include, what to record. As usual, they were asked to reach an agreement about a unique, co‐created plan to produce the video. The main ideas about the video were: (a) it should contain scenes that refer to the idea of sharing cultures and belonging to the territory; (b) it should include an introduction explaining project activities, so as to give an overview of the process to the viewers; (c) a symbolic scene could be produced by presenting a sequence of takes of LL participants′ smiling faces—to suggest the opportunity for people to be together regardless of their culture or country of origin, overcoming prejudices toward the stranger and exalting the peculiarities of each one; (d) short interviews to LL participants could be included to provide an overview of their experiences and comments about being involved in the LL process.

Further, participants proposed to include in the video a “vocabulary of inclusion,” referring to the words they reckoned as more appropriate to the topic after the discussions held during LL meetings. Indeed, according to Gustavsen (2008), the development of new practices cannot be separated from language development; therefore, the co‐creation of the “Vocabulary of inclusion” is the product of the democratic dialogue that took place in the LL. To gather these words, participants stood in a circle and in turn said a word to be included in this vocabulary, then passing the turn to another member who had to choose another word, and so on. The emerging words were: exchange, friendship, discovery, entanglement, network, equal opportunity, exploration, added value, interaction, commitment, together, dialog, union, storytelling, dreams, desire, shared experience, community, openness, doors, international, knowledge, comparison, connection, codesign, active participation, construction, curiosity, experimentation, values, culture, active listening, knowledge, awareness, process, goal, journey, discovery, evolution, emotion, justice, fair future, civilization, experience, root, growth; they were summarized in a wordcloud to be included in the video.

## 5 Discussion

The *leitmotiv* of the described LL experience was the idea that local and migrant youths can and should reciprocally represent a resource to each other, in a relationship of balanced power, opportunities, and interactions (Pienimäki 2021; Sampedro and Camarero 2018). Due to this, LL was chosen as the methodology to be adopted due to it allowing the development of social innovations (European Commission, 2006; Piotrmill 2023) by offering a physical and relational space where cocreation processes could allow participants to produce new ideas, service, and social infrastructures (Butkevičienė et al. 2021; Dekker et al. 2020) aimed at modifying real‐life (un)balances and habits (Kareborn and Stahlbrost 2009; Dell'Era and Landoni 2014; Følstad 2008). In addition, based on them encouraging interaction and confrontation between stakeholders and citizens who have a common goal but different cultures, representations, and experiences (Pataki et al. 2023a), LL simultaneously represented the methodology but also one of the main results itself (Dekker et al. 2020; Schuurman and De Marez 2012).

Indeed, within the framework of LL meetings, the activities were set up as processes of thought which included the development of a concrete vision and transformative strategies (Andersen et al. 2010; Dell'Era and Landoni 2014; Pataki et al. 2023a) with respect to the topics of interest, starting with sharing participants' personal experiences, perceptions, and needs (Bekker and Long 2000; Pataki et al. 2023a; Viswanathan et al. 2004) according to them being native citizens, citizens having a migratory background, and/or stakeholders working on social inclusion and migratory processes issues. Several elements were highlighted by the participants as critical to the promotion of more inclusive social processes: (a) rethinking language so that it could become more inclusive (see the “Vocabulary of inclusion”); (b) giving centrality to the migrant youths and to the storytelling of their experiences, to valorize them; (c) adopting an attitude of curiosity toward and listening of others—even more if they are bearers of different culture, ethnicity, religion, and so forth; (d) deconstructing implicit and explicit prejudices and fighting discriminations; (e) recognizing everyone′s needs and the possibility of mobilizing personal and collective resources to satisfy them. The whole LL group worked jointly to carry out activities towards a common goal (Dell'Era and Landoni 2014), that is, to generate positive changes and reduce the risks of migrant youths' exclusion from their community social life. Prejudices (both implicit and explicit ones) and discrimination of which migrants had been victims were discussed, as well as the training of social workers who provide services, the difficulties linked to language and cultural barriers, the presence of mediators in reception facilities, and the possibility of narrating one's own life story.

This way of working together allowed participants to experience LL meetings in a climate of openness and reciprocal trust, in which everyone felt safe and comfortable in expressing their opinion regardless of their age, role in the territorial community, or gender, making it possible for cultural drivers that connect citizens to emerge (Geatan and Allam 2017). This allowed the circulation of different knowledge from heterogeneous experiences and backgrounds, supporting the group's ability to address shared issues and face common challenges based on its own capacities and ideas (Hooli et al. 2016) as well as on responsibility‐taking and ‐sharing processes (Procentese et al. 2011; Procentese and Gatti 2019b). In this way, more structured knowledge was able to meet naive ones, as well as the voice of those who had experienced discrimination firsthand. This represented one of the community‐related innovative aspects of the LL, that is, it allowed people who perform different roles in the territorial community and who have different backgrounds to meet, share their viewpoints, and activate processes of co‐construction of different ways of living together within communities (Karlsen and Larrea 2014; Pataki et al. 2023a; Procentese et al. 2011). Overall, across LL meetings there was a shift from conversations that were not very rich or in which only part of the group participated in conversations that were rich and in which the whole group felt able to have a say (Dell'Era and Landoni 2014). This also allowed LL participants to feel part of a cohesive group whose members shared the same views about the relationships between local and migrant youths as well as the same interest in spreading this vision in their community of belonging.

The main changes emerged during the LL experience were related to empowerment processes, knowledge development, and skills acquisition, resuming the key principles of the methodology (CoreLabs 2007; Karlsen and Larrea 2014) and the framing of LLs as open community spaces where collaboration, transformation, and empowerment can take place (Kareborn and Stahlbrost 2009; CoreLabs 2007; Hooli et al. 2016; Schwandt et al. 2007). Specifically, young citizens developed further planning skills, as well as a more aware perspective about social inclusion processes and experiences; stakeholders and referents of local associations were able to listen to the experiences and perspectives of the young citizens who directly experienced social inclusion or exclusion processes and enrich their present and future plans by incorporating them. Further, all the involved actors developed a stronger sense of resourcefulness, so they all started suggesting activities, ideas, and initiatives to be jointly planned according to their roles (Bartels and Wittmayer 2014; Hooli et al. 2016; Huxham 2003; Orefice 2006). Indeed, the implemented path promoted the development of critical reasoning, the increase in problem solving skills, as well as the ability to work in a group, enhancing local communities' capacities to address and solve challenges internally (Hooli et al. 2016).

Further, another change stemming from the LL experience refers to the strengthening of the social network in the local community. Indeed, the activities and exchanges of ideas and perspectives within LL meetings allowed participants to reckon that there were other groups and individuals dealing with the same topics and within the same perspectives in their local community. Furthermore, the processes and dynamics involved in LL activities and experiences themselves allowed participants to develop stronger reciprocal trust (Kareborn and Stahlbrost 2009; Chesbrough 2003; Chesbrough and Appleyard 2007). This brought about the rise of synergic activities and initiatives to be implemented in the building on their LL experience, ending in a process of community development and strengthening which is still ongoing (Kareborn and Stahlbrost 2009; Elliott 1991; Francescato et al. 2002).

Moreover, the side advantage of these initiatives was also that they represented the first, spontaneous attempts for LL participants to foster different representations and opportunities about living together in the territorial community and about the relationships and interactions between local and migrant youths (Arcidiacono et al. 2007; Juvonen et al. 2019; Pienimäki 2021; Procentese et al. 2011; Sampedro and Camarero 2018). That is, the described LL experience fostered processes of capacity‐building, relationships creation and maintenance, as well as empowerment for the individuals taking part in the meetings but also for the target community as a whole—consistently with an ecological approach (Bronfenbrenner 1979; Lewin 1951). Therefore, even though social change was not concretely visible and detectable at the end of the LL path itself, these processes of empowerment, capacity building, and community building and strengthening represented fruitful seeds of change which can allow fairer responsibility‐taking and ‐sharing processes in the long term (Procentese and Gatti 2022; Procentese et al. 2019). Indeed, one of the core principles of PAR is that researchers have to “become involved in cycles of defining problems, gathering and sharing information, determining actions and studying what comes of those actions” (Kidd et al. 2018, 77); therefore, changes stemming from PAR can also be compounded by social processes slowly unfolding in how community members share places, relate to each other, and manage common issues and responsibilities, up to modifying the social structures and beliefs that maintain exclusion and inequity processes (Kidd et al. 2018).

Overall, the LL represented a territorial agora (Karlsen and Larrea 2014) in which citizens having different backgrounds and roles could meet and match, starting a process of reciprocal knowledge aimed at developing joint projects involving local associations and interested youths, so that different viewpoints and skills were put together towards a common goal (Bekker and Long 2000; Pataki et al. 2023a; Viswanathan et al. 2004); the democratic approach characterizing this agora allowed participants to produce sustainable ideas valuing everyone′s contribution (Dell'Era and Landoni 2014; Elliott 1991; Francescato et al. 2002; Nowotny et al. 2001). What emerged showed that living in a territorial community hosting citizens having migratory background is not necessarily a premise for a process of mutual cultural contamination (Arcidiacono et al. 2017); therefore, the development of shared projects and visions at the end of the LL represents a relevant result allowing the community to further work together towards common aims and through joint, synergic actions. As to this, the co‐created results emerging from LL experience can represent a concrete path towards the community jointly implementing local changes (Dekker et al. 2020; Schwandt et al. 2007). That is, by fostering active involvement in the detection of potential solutions to local problems and responsibility‐taking and ‐sharing processes, the LL promoted processes of community building based on an active and engaged citizenship (Procentese and Gatti 2022; Zaff et al. 2010). For example, in the specific case of the described LL, participants decided to work together towards planning the joint implementation of the projects they co‐created during the last meetings—which was aimed at achieving more inclusive communities for youths regardless of their ethnic and cultural background. This can represent a first practical implication of this LL—tightly linked to its nature and main pillars.

Further implications stemming from this experience are related to LL broadly representing innovative paths able to promote new opportunities for encounters among citizens expressing and representing different interests and roles as to their community of belonging (Piotrmill 2023). Based on this, they can represent incubators of generative and cocreative relationships and social processes, where continuous dialogue and reflexivity are meant as key elements (Kareborn and Stahlbrost 2009; Karlsen and Larrea 2014) able to bring citizens and local stakeholders towards shared processes of community building. Indeed, LL shaped as a space of reciprocal acknowledgment of individual, community, and territorial resources and power, as well as of the mutual competencies and tools needed to generate social innovation processes. Making youths' and stakeholders' different instances, perspectives, and missions emerge and dialogue facilitated mutual understanding and the acknowledgment of the perspectives of young citizens of different ethnicities and holding different cultural backgrounds, and enabled the recognition of different languages, the acquisition of intercultural communication and agency skills. The shared vision emerging could represent the basis for the development of collaborative and sustainable shared projects (Dell'Era and Landoni 2014). Therefore, LLs can represent a valuable tool for research and intervention—especially when they are meant to fit within the PAR approach (Orefice 2006)—but also for policy makers and local stakeholders (e.g., associations) interested in promoting participatory and co‐developed social innovations aimed at improving individual and collective life conditions in their communities (e.g., Bifulco et al. 2017; Lehmann et al. 2015; Voytenko et al. 2016); such processes could produce benefits for public and private organizations as well as for citizens (Hossain et al. 2019).

### 5.1 Limitations and Future Directions

The results from this study provide insights into (a) how to foster innovative, participatory social inclusion processes based on reciprocity and acknowledgment (Pienimäki 2021; Sampedro and Camarero 2018), for the promotion of local participation, individual and shared responsibility‐taking processes, and sense of community, and (b) the potential of LLs as incubators of participatory and collaborative processes (Kareborn and Stahlbrost 2009; CoreLabs 2007; Hooli et al. 2016) aimed at community building. However, it should be mentioned that the citizens involved were only a small, nonrepresentative group and that the process and the results are to be understood with reference to the specific context where they were carried out and to its characteristics.

Nevertheless, despite these limitations, the present study meets trustworthiness, fairness, and transferability criteria (Guba 1981; Lincoln and Guba 1988; Schwandt et al. 2007) and therefore provides meaningful insights into future research and interventions. Furthermore, due to the LL relying on an iterative process aimed at producing final results which clearly stem from the implemented interactions and collaborations—and are not set before LL unfolding (Dekker et al. 2020; Schwandt et al. 2007), the described processes, methods, and tools (see Table 1 for a summary) can represent a reliable and valuable path to be played out also in different contexts and referring to different topics or social issues—eventually, also adapting it to researchers' and community's needs (e.g., long‐term sustainability of citizens' engagement, availability of the needed resources). This rather represents a strength—consistently with a constructivist and naturalistic approach (Guba and Lincoln 1994; Schwandt et al. 2007)—since modern socio‐cultural and political phenomena (e.g., migratory processes, climate crisis) represent cross‐cutting issues impacting modern local communities all over the world, yet locally shaping based on context political, economic, social, physical, and environmental peculiarities (e.g., Moyano et al. 2022; Prati et al. 2017; United Nations 2016).

## Ethics Statement

The authors declare that the research is conducted ethically, responsibly, and legally; the results are reported honestly; the submitted work is original and not (self‐)plagiarized; funding sources and conflicts of interest are disclosed. The study was conducted in accordance with the Declaration of Helsinki and approved by the Ethical Committee of Psychological Research of the University of Naples Federico II (protocol code 1/2022, date of approval January 17, 2022).

## Conflicts of Interest

The authors declare that there are no potential conflicts of interest with respect to the study, authorship, and publication of this article.

## Data Availability

Data sharing is not applicable to this article as no new data were created or analyzed in this study.
